# P-909. Management of Diabetic Foot Osteomyelitis Following Surgical Intervention: Trends from an Academic Medical Center

**DOI:** 10.1093/ofid/ofaf695.1115

**Published:** 2026-01-11

**Authors:** Sean Harford, Majd Alsoubani, Kap Sum Foong, Maher Jafar, Geneve Allison

**Affiliations:** Hartford Healthcare Medical Group, Glastonbury, CT; Tufts Medical Center, Boston, Massachusetts; Tuft Medical Center, Tufts University School of Medicine, Boston, MA; Tufts Medical Center, Boston, Massachusetts; Tufts Medical Center, Boston, Massachusetts

## Abstract

**Background:**

The Infectious Diseases Society of America (IDSA) published new guidelines for the management and treatment of diabetic foot infections in 2023. The guidelines recommended consideration of up to three weeks of antibiotic therapy for diabetic foot osteomyelitis (DFO) after minor amputation even with positive culture or histology of bone margins. Recommendations however were conditional with a low certainty of evidence. The aim of this study is to evaluate management trends of antimicrobial therapy and factors associated with adherence to these recommendations at an academic medical center.

**Table 1:**
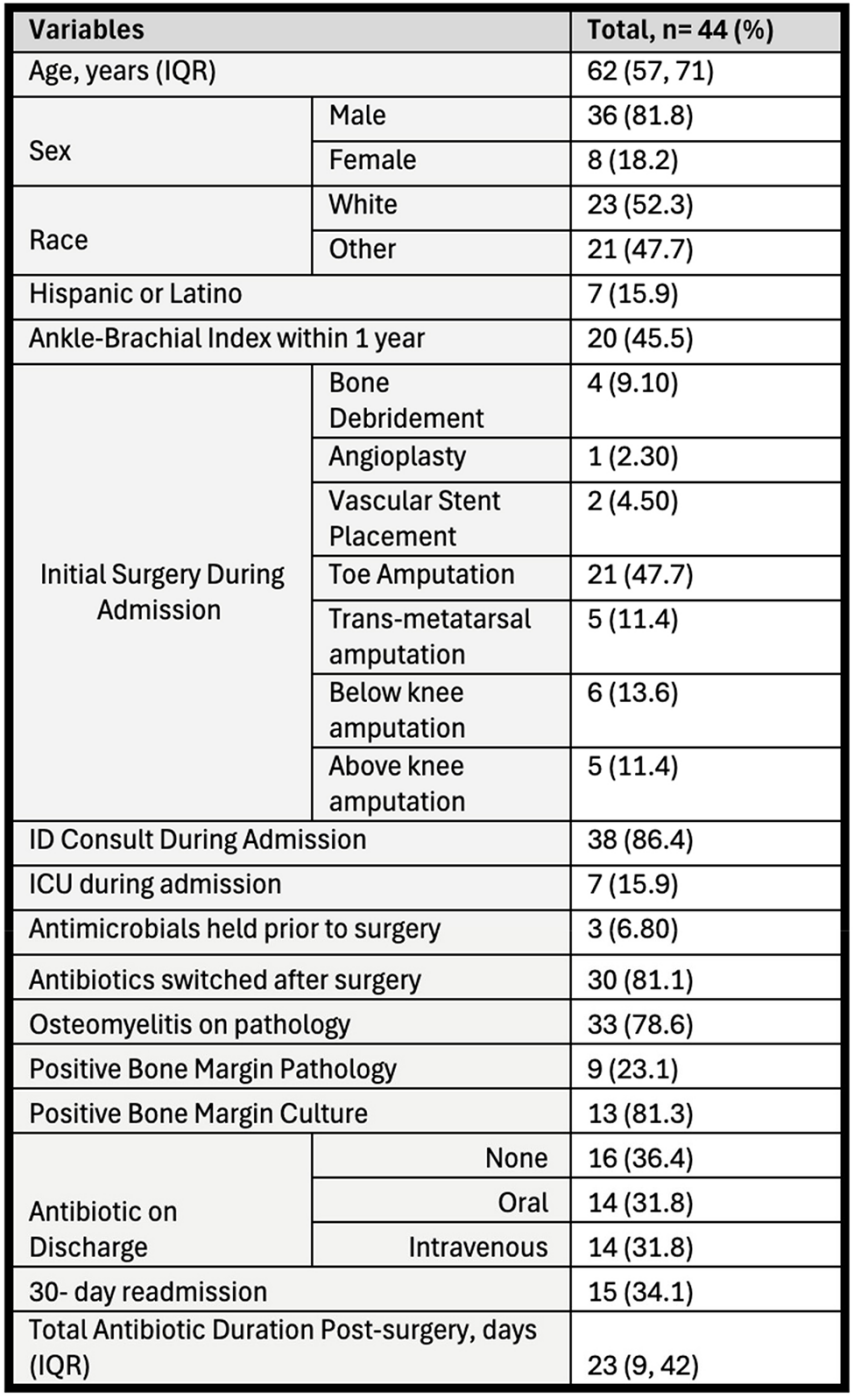
Demographic and Outcomes Baseline Data Among Inpatients With Diabetic Foot Osteomyelitis. IQR, Interquartile Range

**Table 2:**
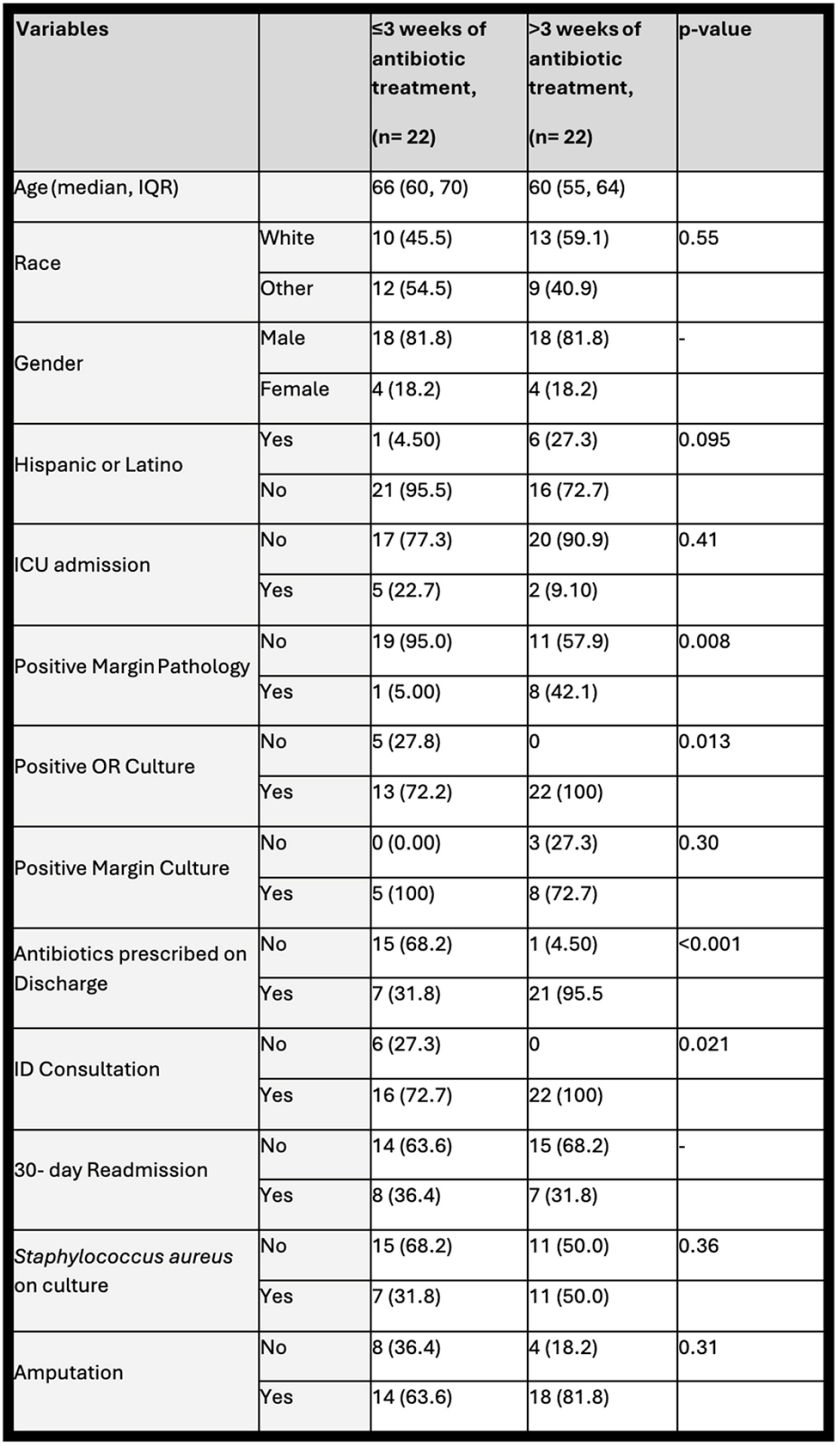
Factors Associated With Antibiotic Treatment Duration Among Inpatients with Diabetic Foot Osteomyelitis.

**Methods:**

This is a retrospective study of adult inpatients with a diagnosis of diabetic foot infection requiring surgical intervention by the vascular surgery service between January 1, 2023 and 31 December 31, 2023. The primary outcome was adherence to the IDSA DFO antibiotic duration recommendations. Demographics and clinical data were collected. Categorical variables were summarized as counts and percentages; continuous variables as medians with interquartile ranges. We assessed group differences using Chi-square and Mann-Whitney tests.

**Results:**

We identified 44 patients with DFO who underwent surgical debridement. As depicted in Table 1, most patients were male, white, underwent toe amputations as initial surgery, had antibiotics started prior to surgical intervention, and received ID consultation. Median age was 62 and the median antibiotic duration post-surgical intervention was 23 days.

Patients with positive bone margin pathology, positive surgical cultures, ID consultation, or discharged on oral antibiotics were more likely to receive a longer antibiotic course (>3 weeks) (Table 2). No differences were observed in readmission, positive margin cultures, amputation outcomes, ICU stay, or *Staphylococcus aureus* isolation.

**Conclusion:**

Despite new IDSA guidelines recommending shorter antibiotic courses, prolonged treatment remains common for DFO with positive bone margins. Further research is needed to examine outcomes associated with shorter-duration therapy for DFO.

**Disclosures:**

All Authors: No reported disclosures

